# Point Set Denoising Using Bootstrap-Based Radial Basis Function

**DOI:** 10.1371/journal.pone.0156724

**Published:** 2016-06-17

**Authors:** Khang Jie Liew, Ahmad Ramli, Ahmad Abd. Majid

**Affiliations:** School of Mathematical Sciences, Universiti Sains Malaysia, 11800 Minden, Penang, Malaysia; Beijing University of Technology, CHINA

## Abstract

This paper examines the application of a bootstrap test error estimation of radial basis functions, specifically thin-plate spline fitting, in surface smoothing. The presence of noisy data is a common issue of the point set model that is generated from 3D scanning devices, and hence, point set denoising is one of the main concerns in point set modelling. Bootstrap test error estimation, which is applied when searching for the smoothing parameters of radial basis functions, is revisited. The main contribution of this paper is a smoothing algorithm that relies on a bootstrap-based radial basis function. The proposed method incorporates a *k*-nearest neighbour search and then projects the point set to the approximated thin-plate spline surface. Therefore, the denoising process is achieved, and the features are well preserved. A comparison of the proposed method with other smoothing methods is also carried out in this study.

## Introduction

The input from 3D scanning devices will often produce noise in the positions of vertices. A variety of denoising algorithms have been proposed to address this issue. The term denoising is literally interpreted as the process of removing noise from the data. From the existing literature, this term is widely used interchangeably with smoothing. Basically, denoising algorithms can be divided into mesh denoising and point set denoising. The main difference between point set denoising and mesh denoising is the absence of connectivity in the former. Point set denoising is the main focus of this study.

Point set denoising deals with points in Euclidean space, whereas mesh denoising uses a mesh representation. Point sets are gaining attention as a representation of models in computer graphics because of the invention of accurate and affordable scanning devices that generate a point set. The moving least squares (MLS) method is widely used in point set denoising. The MLS method was proposed by [1] for smoothing and interpolating data. A detailed survey on the MLS method is provided in [2]. The rigorous mathematical concepts and proofs involved are described in [3], and the author shows that the local error of the MLS approximation is bounded. However, the basic mathematical concept underlying MLS is introduced here. Let a new point *q*_*i*_ move along a direction normal to a considered point *r*_*i*_. In the local frame of point sets, a value of *q*_*i*_ is found that will minimise the weighted distance between the points and the tangent plane *H* as

$$\begin{array}{c} \text{min}\sum_{j=1}^{n}D\left(p_{j}\right)\theta \left(p_{j},q_{i}\right) \end{array}$$

where *D*(*p*_*j*_) is the nearest distance from the points *p*_*j*_ to the tangent plane on which *q*_*i*_ lies and *θ*(*p*_*j*_, *q*_*i*_) is the weight-based function of the distance from *p*_*j*_ to *q*_*i*_ and is positive. Then, the polynomial fit *f*_*j*_, which is a *n*-order polynomial, is found such that it minimises the following equation

$$\begin{array}{c} \text{min}\sum_{j=1}^{n}{\left(d\left(f_{j}-p_{j}\right)\right)}^{2}\theta \left(p_{j},q_{i}\right) \end{array}$$

Then, the considered point *r*_*i*_ is moved such that it lies on the polynomial surface defined.

In [4], the point set that is generated from 3D scanning devices may be contaminated by noise; therefore, the original point set is replaced by the approximated point set, which is obtained from the MLS surface. The authors show that the approximation error is bounded. Therefore, depending on the point set density, the numbers of point sets can be either up- or down-sampled to control the approximation error. [5] further discuss the MLS surface and analyse the projection procedure of points on the MLS surface. [6] propose another projection procedure that uses a locally optimal projection operator to create an approximate surface from a point set. This operator is a parametrisation-free projection that does not rely on the local parametric representation, such as the local normal or local plane. It can be used in the pre-process stage of surface reconstruction and in denoising or eliminating outliers. [7] derives a sampling condition and provides the accompanying proof. The sampling condition ensures that the normal approximation is well defined within the neighbourhood of the sample, which will eventually lead to a smooth MLS surface.

[8] propose a signal processing method that uses Fourier analysis for point set surface processing. They claim that many common filter operations can be computed efficiently, though the pre-processing of this method is costly. [9] introduce an algorithm to construct high-quality triangulation from a point set surface that is capable of smoothing the noisy data but still uses the MLS surface as the underlying representation. Their method has some limitations because it has a high computational cost, cannot preserve the sharp features, and may have coarser triangulations. [10] incorporate a statistical method to filter the noisy data by suppressing noise of different amplitudes. Their approach is also able to detect and diminish the outliers. However, their method does not focus on sharp feature preservation, though it can be applied to a very large point set model. [11] propose a non-local neighbourhood filtering for denoising range scans before further processing while having the ability to preserve fine sharp features.

[12] extend a similar concept and approach to bilateral filtering to that provided in [13] for mesh smoothing to point set smoothing that can preserve sharp features and edges. [14] note that conventional planar MLS will have unstable projection issues under a low sampling density; therefore, they propose directly fitting higher-order algebraic surfaces that are able to handle sharp features, such as a spherical fit. However, the noise issue is not mentioned, and the smoothing procedure is thus not discussed in their work. This work is then further generalised in [15], where a parameter of curvature control in fitted spheres is introduced that is able to remove noise without losing the surface structure. [16] use the statistical method bootstrapping to determine the test error estimation and then apply the fitted bootstrap surface for denoising purposes. Their method is able to detect and handle sharp features.

In this paper, the bootstrap test error estimation of a radial basis function in [17] will be applied for denoising a point set model that is corrupted by noise. There is not much associated literature on applying bootstrap-based radial basis functions in smoothing the point set model; hence, a smoothing algorithm is proposed. The mathematical background for a radial basis function, bootstrap method, and *k*-nearest neighbour search method will be introduced briefly. The methodology for smoothing using thin-plate splines will be described in the section Materials and Methods. Section 3 will present the graphical results and the validation for the proposed method. In the Discussion section, a discussion based on the results obtained from the Results section will be presented. Finally, we will present our conclusions in the Conclusion section.

## Materials and Methods

### Radial Basis Function

The use of radial basis functions (RBFs) for surface representation dates back to the Hardy’s paper [18]. In 1975, another RBF, the thin plate spline, was discovered by [19], and it is an example of a global basis function. Duchon mentions that RBF is invariant to translations and rotations of coordinate systems over $R^{n}$. In addition, it provides a solution for the scattered data interpolation problem in multi-dimensional spaces in the form of polynomials. Generally, the interpolation problem can be described as follows. Given a set of distinct data points (also known as nodes) $X={\left\{x_{i}\right\}}_{i=1}^{N}\subset R^{n}$ and a set of function values ${\left\{f_{i}\right\}}_{i=1}^{N}\subset R$, find an interpolant $s:R^{n}\to R$ such that

$$\begin{array}{c} s\left(x_{i}\right)=f_{i},\;i=1,\ldots ,N \end{array}$$ (1)

Concepts and works related to RBF can be found in [20–23]. The form of a general RBF is as follows:

$$\begin{array}{c} s\left(X\right)=p\left(X\right)+\sum_{j=1}^{N}\lambda_{j}\phi (|X-X_{j}\left|\right) \end{array}$$ (2)

The choices for RBF include splines that are polyharmonic, Gaussian *ϕ*(*r*) = *e*^−*cr*^2^^, multiquadric $\phi \left(r\right)=\sqrt{r^{2}+c^{2}}$, and inverse multiquadric $\phi \left(r\right)=\frac{1}{\sqrt{r^{2}+c^{2}}}$. The value *c*, which can be a Gaussian, multiquadric, or inverse multiquadric spline, is a user-defined value. A good choice for a polyharmonic spline for fitting a smoothing function of two variables is the thin-plate spline *ϕ*(*r*) = *r*^2^ *log*(*r*), which has *C*^1^ continuity, whereas for fitting a smoothing function of three variables, good choices are the biharmonic spline *ϕ*(*r*) = *r* and triharmonic spline *ϕ*(*r*) = *r*^3^. The biharmonic and triharmonic splines have *C*^1^ continuity and *C*^2^ continuity, respectively. The Gaussian spline is mainly used for neural networks, whereas the multiquadric spline is used for fitting topographical data. The experimental results use the Gaussian spline and compactly supported piecewise polynomials for surfaces fit onto point clouds, which will have surface artefacts introduced because of the lack of extrapolation across the holes [21].

Suppose that we want to interpolate the data of two variables and set the polynomial *p* as the linear form; then, the thin-plate spline interpolant is $s:R^{2}\to R$ such that:

$$\begin{array}{ccc} s(x_{i},y_{i}) & = & a_{1}+a_{2}x_{i}+a_{3}y_{i}\\ & & +\sum_{j=1}^{N}\lambda_{j}\phi \left(\sqrt{{\left(x_{i}-x_{j}\right)}^{2}+{\left(y_{i}-y_{j}\right)}^{2}}\right)\\ & = & f_{i} \end{array}$$

where *ϕ*(*r*) = *r*^2^ *log*(*r*).

Because there are *N* + 3 unknowns, three additional solution constraints are added such that

$$\begin{array}{c} \sum_{j=1}^{N}\lambda_{j}=\sum_{j=1}^{N}\lambda_{j}x_{j}=\sum_{j=1}^{N}\lambda_{j}y_{j}=0 \end{array}$$

which yield the linear system written in (*N* + 3) × (*N* + 3) matrix form as follows:

$$\begin{array}{c} \left[\begin{array}{ccccccc} 0 & 0 & 0 & 1 & 1 & \cdots & 1\\ 0 & 0 & 0 & x_{1} & x_{2} & \cdots & x_{N}\\ 0 & 0 & 0 & y_{1} & y_{2} & \cdots & y_{N}\\ 1 & x_{1} & y_{1} & \phi (r_{1,1}) & \phi (r_{1,2}) & \cdots & \phi (r_{1,N})\\ \vdots & \vdots & \vdots & \vdots & \vdots & & \vdots \\ 1 & x_{N} & y_{N} & \phi (r_{N,1}) & \phi (r_{N,2}) & \cdots & \phi (r_{N,N}) \end{array}][\begin{array}{c} a_{1}\\ a_{2}\\ a_{3}\\ \lambda_{1}\\ \vdots \\ \lambda_{N} \end{array}]=[\begin{array}{c} 0\\ 0\\ 0\\ f_{1}\\ \vdots \\ f_{N} \end{array}\right] \end{array}$$

where $\phi \left(r_{i,j}\right)=\phi \left(\sqrt{{\left(x_{i}-x_{j}\right)}^{2}+{\left(y_{i}-y_{j}\right)}^{2}}\right)$.

The matrix form can be further simplified as

$$\begin{array}{c} \left[\begin{array}{cc} 0 & P^{T}\\ P & A \end{array}][\begin{array}{c} a\\ \lambda \end{array}]=[\begin{array}{c} 0\\ f \end{array}\right] \end{array}$$ (3)

where *P* is the matrix with *i*th row (1, *x*_*i*_, *y*_*i*_), λ = (λ_1_, λ_2_, …, λ_*N*_)^*T*^, *a* = (*a*_1_, *a*_2_, *a*_3_)^*T*^ and *f* = (*f*_1_, *f*_2_, …, *f*_*N*_)^*T*^. By solving the linear system, the values of λ and *a* can be uniquely determined, and the function *s*(*x*, *y*) is derived. It is appropriate to use the direct method to solve the above matrix for a problem with, at most, a few thousand points, that is, *N* < 2000.

If noise is present in the data points, the interpolation condition of Eq (1) is strict. Therefore, the condition is to be relaxed to favour smoothness. Let us consider the problem for which we are given nodes ${\left\{x_{i}\right\}}_{i=1}^{N}\subset R^{n}$ and minimise

$$\begin{array}{c} \rho {∥s∥}^{2}+\frac{1}{N}\sum_{i=1}^{N}{\left(s\left(x_{i}\right)-f_{i}\right)}^{2} \end{array}$$

where *ρ* ≥ 0. This problem is known as spline smoothing, and the parameter *ρ* controls the quality of the approximation; in other words, it controls the trade-off between smoothness and fidelity to the data [24]. The solution for this problem is also a RBF of the form given in Eq (2). A smaller value of *ρ* will provide a better approximation and will be an exact interpolation if *ρ* tends to 0. By modifying Eq (3), we have the following:

$$\begin{array}{c} \left[\begin{array}{cc} 0 & P^{T}\\ P & A+\rho I \end{array}][\begin{array}{c} a\\ \lambda \end{array}]=[\begin{array}{c} 0\\ f \end{array}\right] \end{array}$$ (4)

where *I* is an identity matrix. By solving the system of linear equations in Eq (4), we obtain *a* and λ and then plug them into Eq (2). Thus, an approximation of an RBF is obtained.

### Selection of Parameter *ρ* via Bootstrap Method

As given in Eq (4), the *ρ* is known as smoothing parameter that controls the quality of fitting from a set of data points in the presence of noise. As mentioned in the subsection of radial basis function, a smaller value of *ρ* may provide a better approximation for thin plate spline, but it is unknown of how small it can be. Therefore, choosing an appropriate or optimum smoothing parameter *ρ* is essential. A work has been done by us in [17] on searching the optimum smoothing parameter using a bootstrap error estimation method. We will describe our previous work on the bootstrap method and relate it with selection of optimum *ρ* value. With noise-free data points, the *ρ* that is very close to zero is picked as the optimum value for the approximation version of thin plate spline. In the case of noise-free, we can also set *ρ* to zero, that is the points are interpolated and a smooth surface is produced. However, the data points always contaminated by different levels of noise and therefore, it is unsuitable to use *ρ* that is approximately close to zero.

In order to select the optimum parameter *ρ* for surface fitting, we use a statistical method, that is bootstrap method. The bootstrap method is based on repetition of random resampling of the data and averaging the results obtained from each sample. The reuse of the data as a result of repetitive resampling is helpful when the available data are sparse and limited. The sampling procedure is repeated *B* times to produce *B* independent bootstrap sets, *V*^∗*b*^, where *b* = 1, 2, 3, …, *B*. Specifically, we find the optimum smoothing *ρ* parameter by using bootstrap leave-one-out error estimation. The formula for bootstrap leave-one-out error is given as

$$\begin{array}{c} E\hat{r}r=\frac{1}{N}\sum_{i=1}^{N}\frac{1}{n_{C_{i}}}\sum_{b\in C_{i}}\left|f^{*b}\left(x_{i},y_{i}\right)-z_{i}\right| \end{array}$$ (5)

where *C*_*i*_ is the index set of bootstrap sets that does not contain the point *v*_*i*_ and *n*_*C*_*i*__ denotes the size of the set *C*_*i*_. Note that *f*^∗*b*^ in Eq (5) is the approximation scheme of the thin-plate spline in this paper, as shown in Eq (4). To prevent the *n*_*C*_*i*__ equals to the zero, either the large *B* has to be chosen, or the terms in Eq (5) corresponding to *n*_*C*_*i*__’s that are zero is left out. Details regarding the bootstrap method can be found in [16, 25]. Note that *f*^∗*b*^ in Eq (5) is the approximation scheme of the thin-plate spline in this paper, as shown in Eq (2) after solving for *a* and λ in Eq (4).

By using bootstrap leave-one-out error estimation, we test a list of possible *ρ* that fall within the range of *h* value, where the variable *h* is the average distance between two nearest points in a set of points. The *ρ* value that corresponds to the smallest bootstrap leave-one-out error is selected as the optimum value. If values are smaller than optimum value, the noisy data may be overfitted and produce an unpleasant surface. On the other hand, if values are larger than optimum value, the underfit may occur. Therefore, the optimum *ρ* value is the recommended value that can fit the noisy data perfectly.

### Point Set Smoothing Using Bootstrap-based Radial Basis Function

As mentioned in the Introduction section of this paper, the bootstrap error estimation method, specifically the bootstrap leave-one-out error, is implemented here to search for the smoothing parameter *ρ* in the approximation scheme of the thin-plate spline [17]. This approximation scheme is studied and extended in smoothing the point set models that are corrupted by the noisy data. Therefore, in this paper, a new point set smoothing algorithm, which is based on the bootstrap fitted thin-plate spline surface patch, is proposed for denoising the point set. The Stanford bunny and bimba point sets models with noise levels of 0.25 and 0.50 are used to test the proposed smoothing algorithm. Before starting the denoising process, a suitable smoothing parameter *ρ* will be searched for using the method in [17] because different sizes of *k*-nearest neighbourhoods will have different smoothing parameters. Hence, an approximated thin-plate surface patch is produced from the set of sample data points, which is obtained using the *k*-nearest neighbour search method. Next, the set of sample data points is projected on the approximated thin-plate spline surface patch. The updated positions of the data points are used to smooth the noisy point set model. With these required materials, the smoothing algorithm is described as follows:

**Proposed point set smoothing algorithm**

**Input**: Noisy point set model

**Output**: Smoothed point set model

 1. The sample of data points, *S*, with size *N* is uniformly selected from the point set model.

 2. Calculate the global *h* value for the data points *S* with size *N*.

 3. Using the *k*-nearest neighbour search method, a neighbourhood with size *k*_1_ is selected, and the local *h* value of the neighbourhood is then determined.

 4. If the local *h* value is greater than the global *h* value, then the neighbourhood of size *k*_1_ is used; else, the neighbourhood of size *k*_2_, which is larger than *k*_1_, is used.

 5. Fix the bootstrap sets of size *B*. Next, determine the optimum smoothing parameter *ρ* of the thin-plate spline for the size of neighbourhoods *k*_1_ and *k*_2_ by using the method that we proposed in [17].

 6. The principal component analysis (PCA) method discussed in [26] is used to estimate the normal vector for each neighbourhood of data points obtained in Step 4.

 7. Reorient the position of the selected *k*_1_ (or *k*_2_)-nearest data points to the ordinary XYZ axis position using the information from the normal vector from Step 6.

 8. Fit the *k*_1_ (or *k*_2_) data points by using the approximation scheme for the thin-plate spline surface.

 9. Project the *k*_1_ (or *k*_2_) data points on the approximation scheme of the thin-plate spline surface; hence, the updated *k*_1_ (or *k*_2_) data points are now reoriented to the original position, the position before orientation by the normal vector.

 10. The vertices of that particular neighbourhood of data points are updated based on the latest position of data points in Step 9.

 11. Steps 3 to 10 are repeated for another point in *S* until *N* times.

The values *N*, *B*, *k*_1_, and *k*_2_ are user-defined values. The result obtained from the algorithm is compared with a mesh smoothing method, the Humphrey Classes (HC) Laplacian smoothing algorithm, which is an improvement upon the ordinary Laplacian smoothing algorithm. A comparison is also performed with a point set smoothing method on the MLS, which is an algebraic point set surface. For the following section, the smoothing and comparison results are displayed graphically to validate the proposed algorithm.

## Results

The following results are obtained by testing the Stanford bunny and bimba point set models. The OFF files for Stanford Bunny, Bimba, and sphere are available from S1–S9 Datasets.

## Discussion

**Fig 1:**
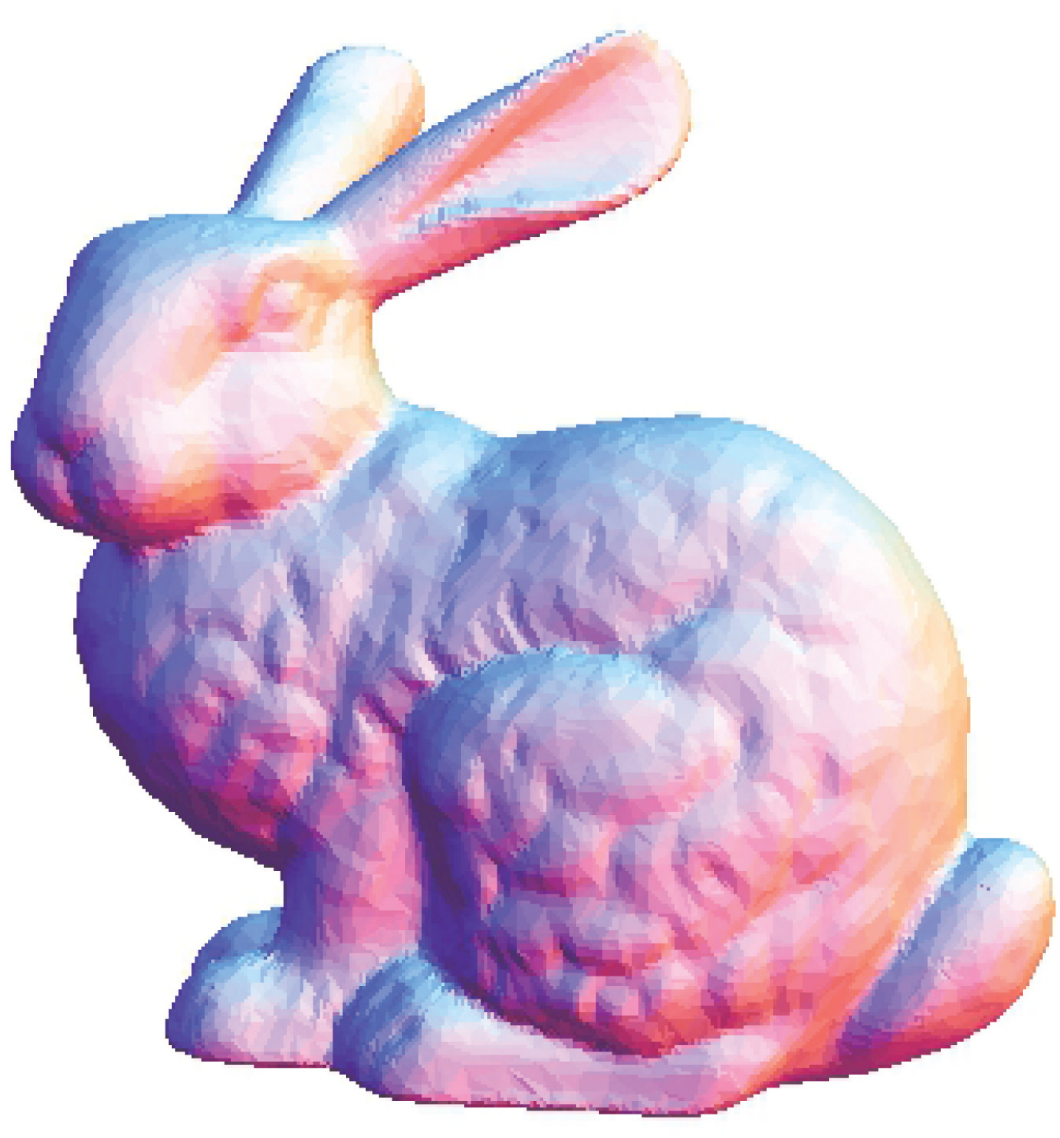
The noise-free Stanford bunny point set model.

**Fig 2:**
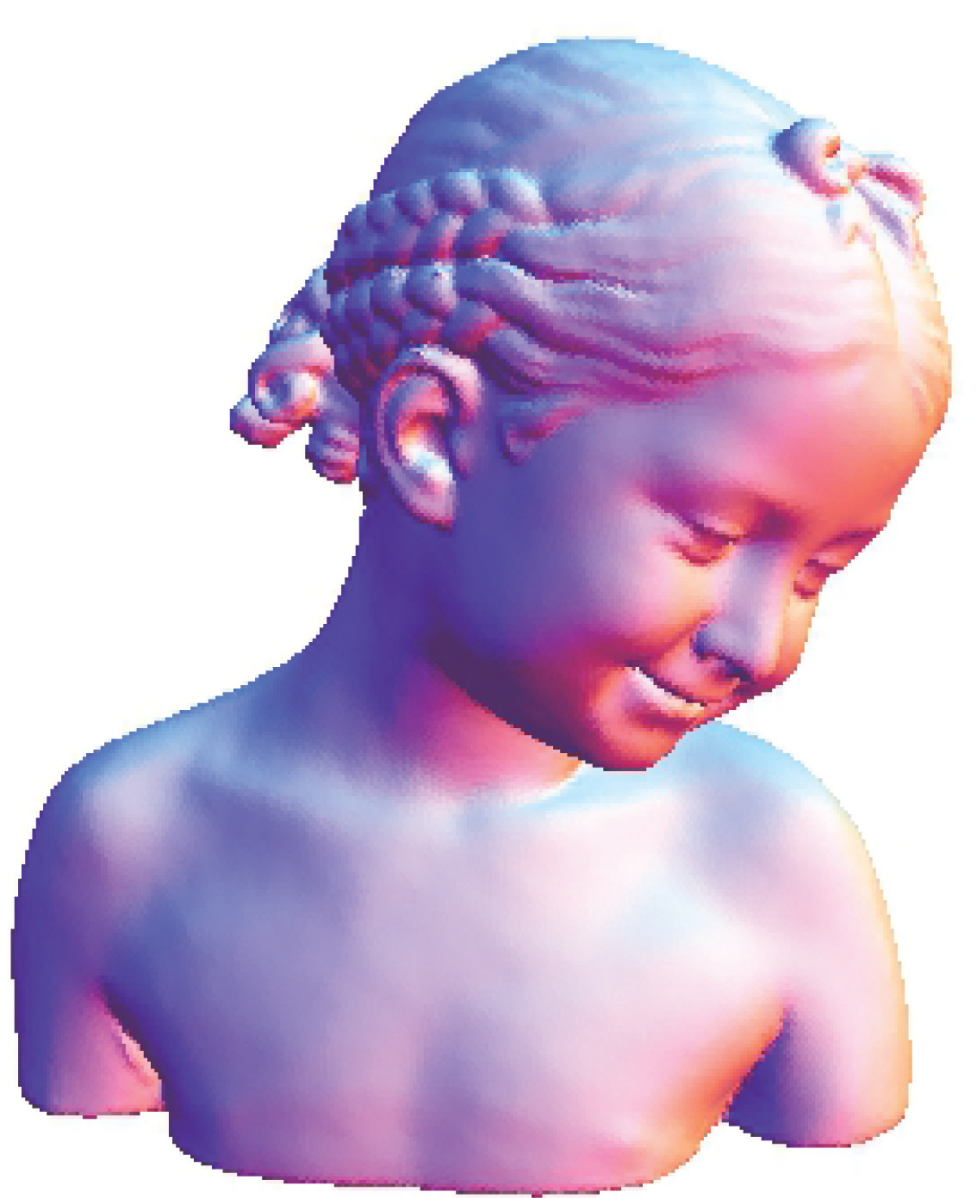
The noise-free bimba point set model.

**Fig 3:**
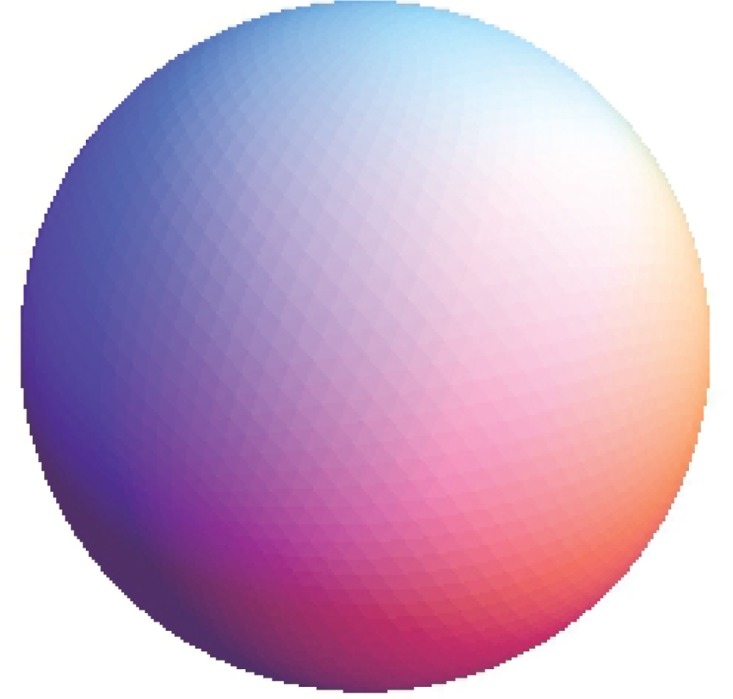
The noise-free sphere point set model.

**Fig 4:**
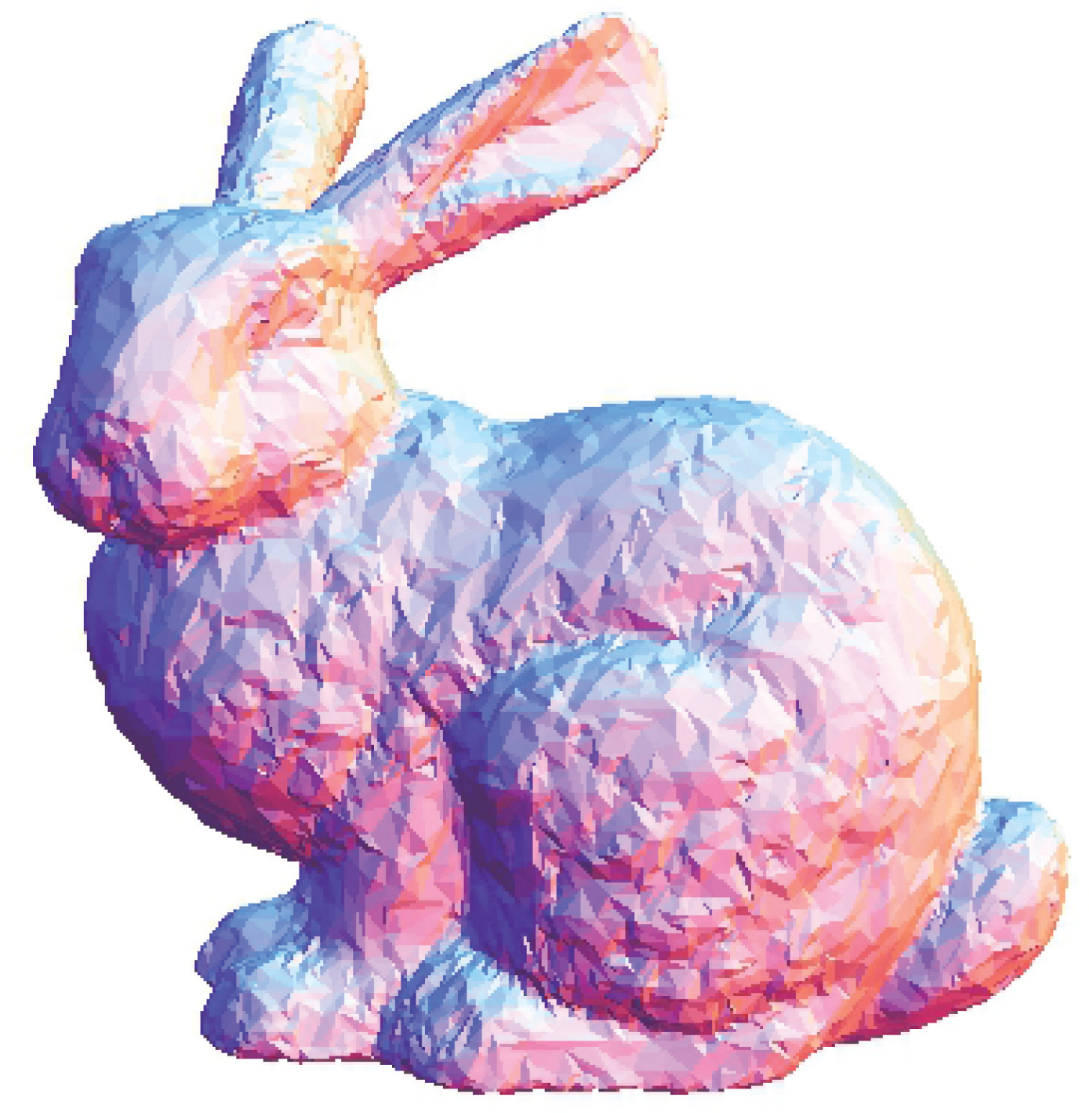
The Stanford bunny point set model with a noise level of 0.25.

**Fig 5:**
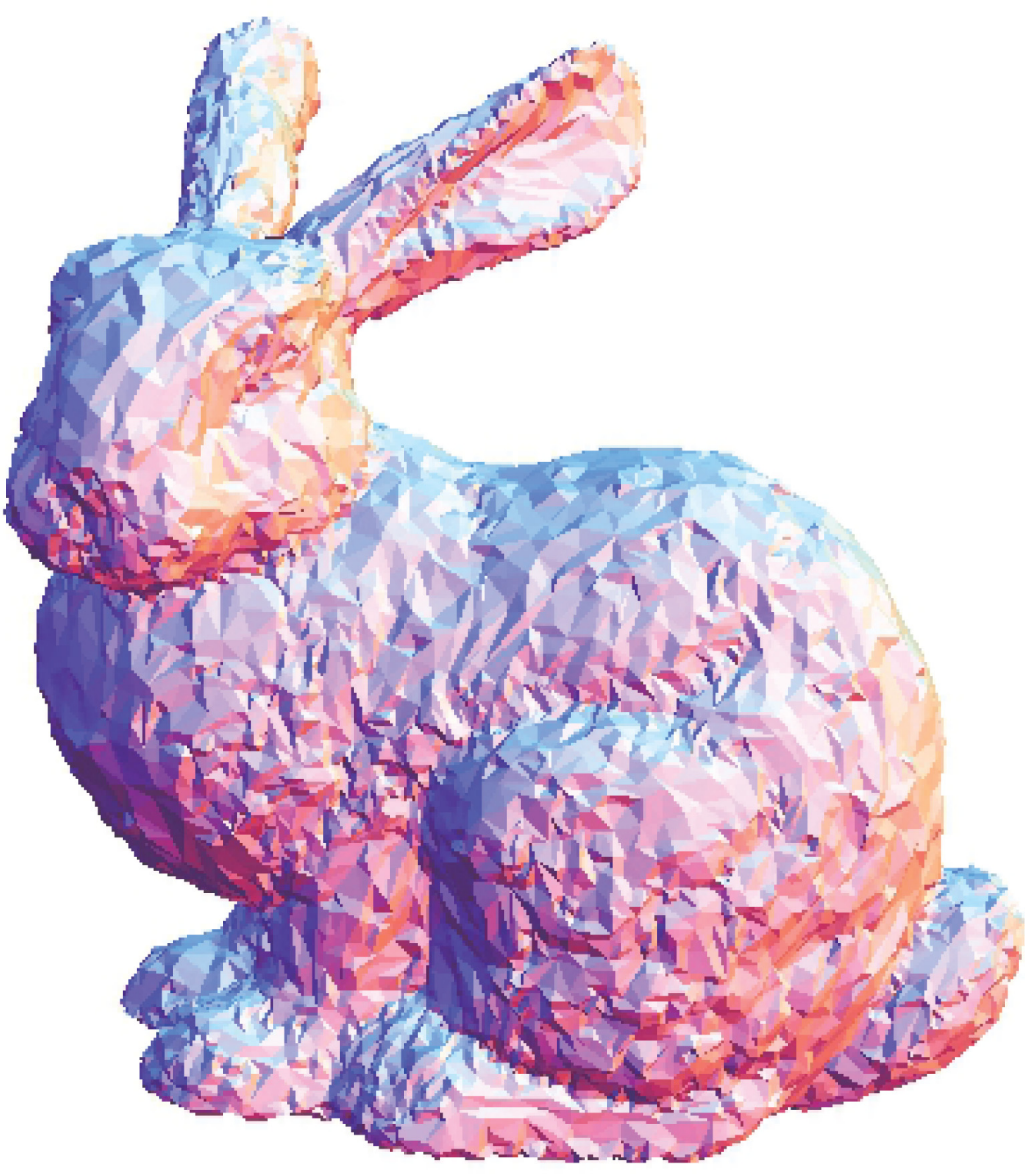
The Stanford bunny point set model with a noise level of 0.50.

**Fig 6:**
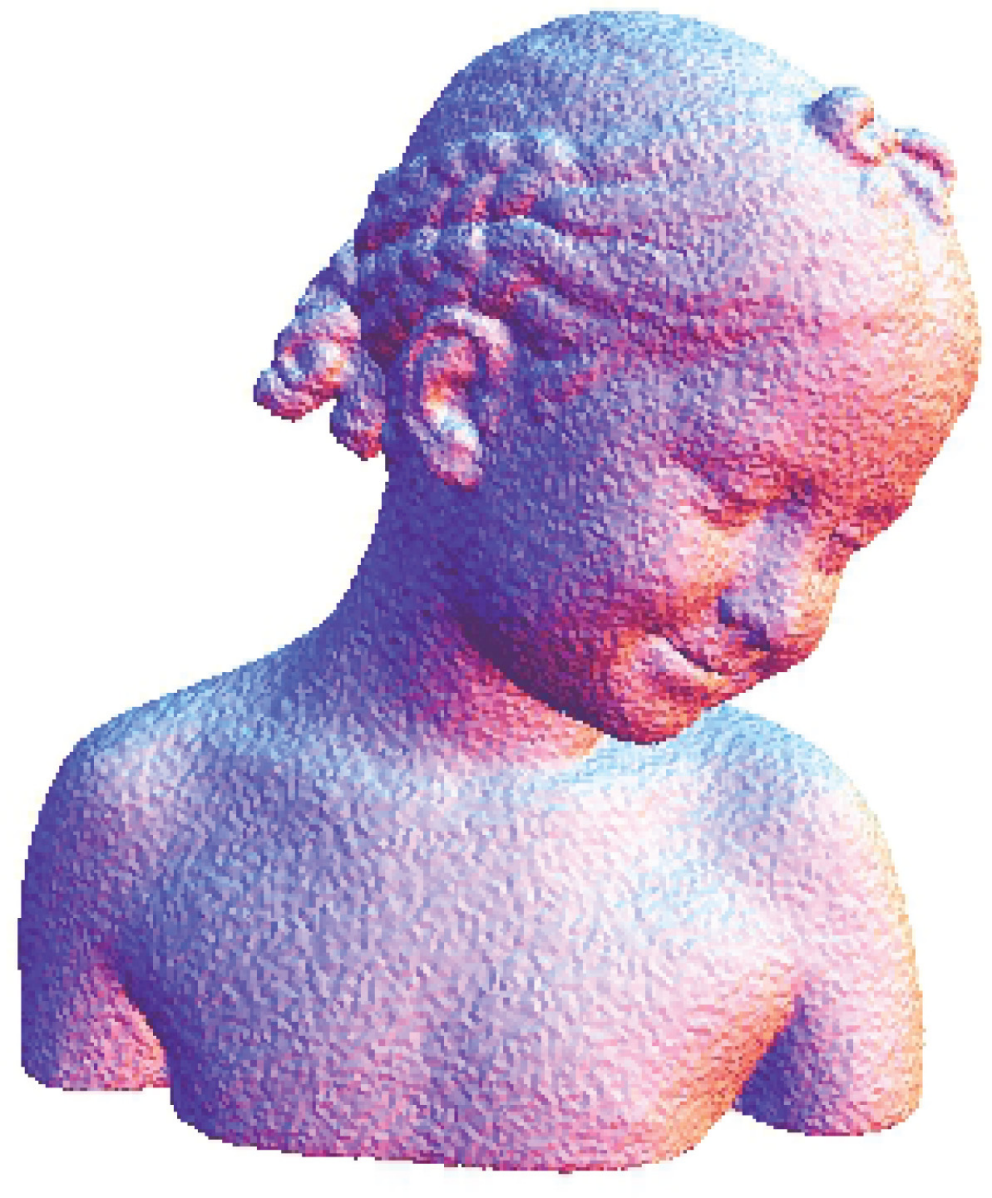
The bimba point set model with a noise level of 0.25.

**Fig 7:**
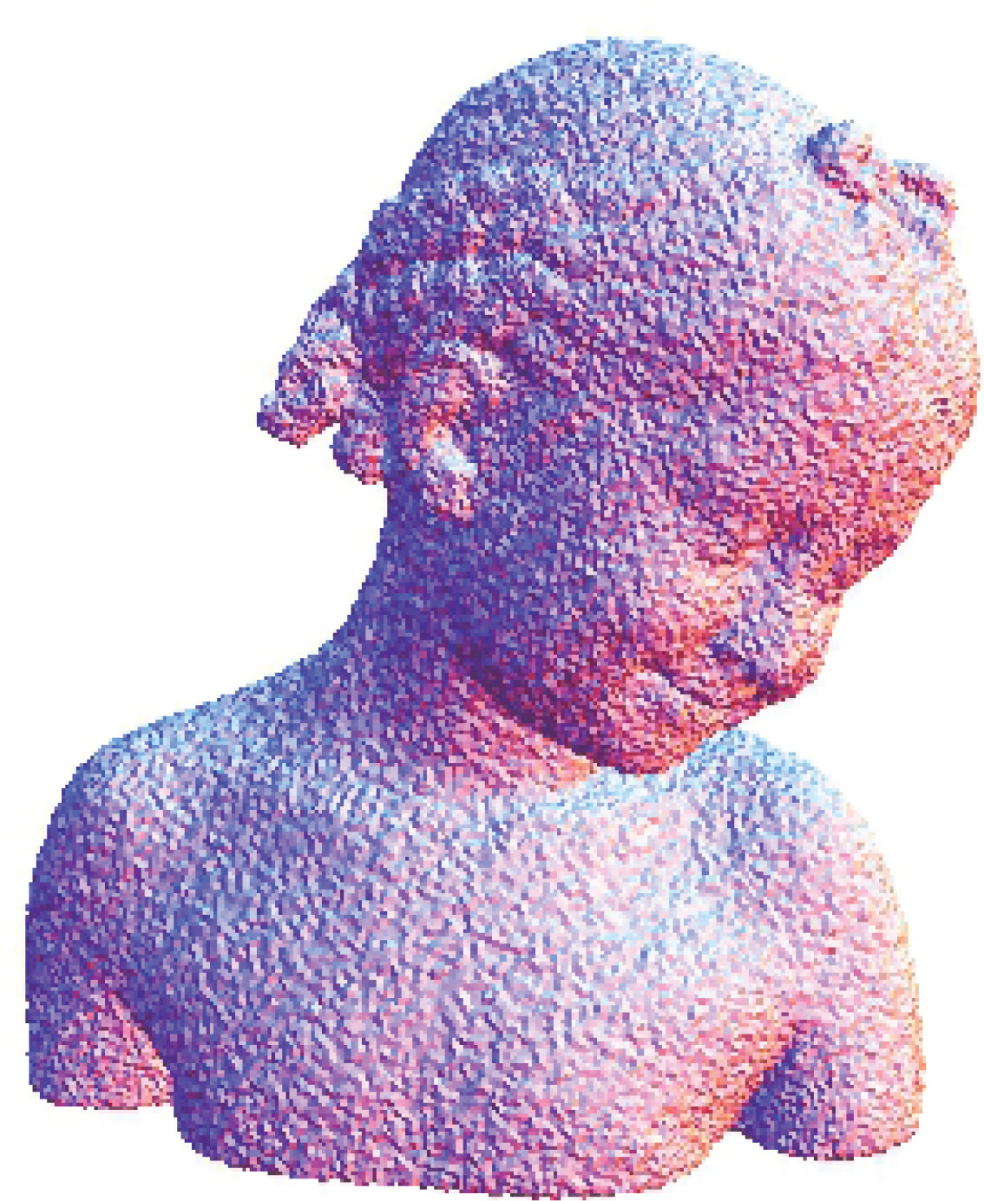
The bimba point set model with a noise level of 0.50.

**Fig 8:**
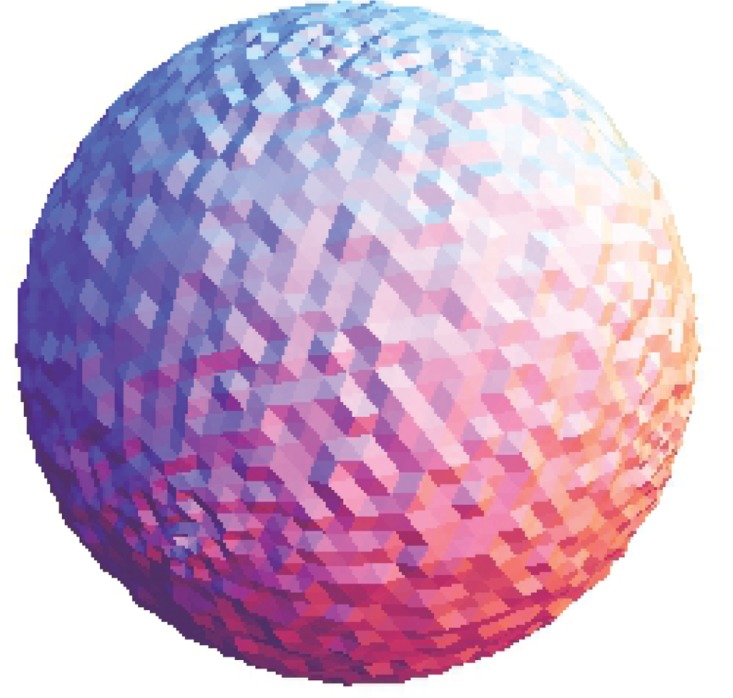
The sphere point set model with a noise level of 0.25.

**Fig 9:**
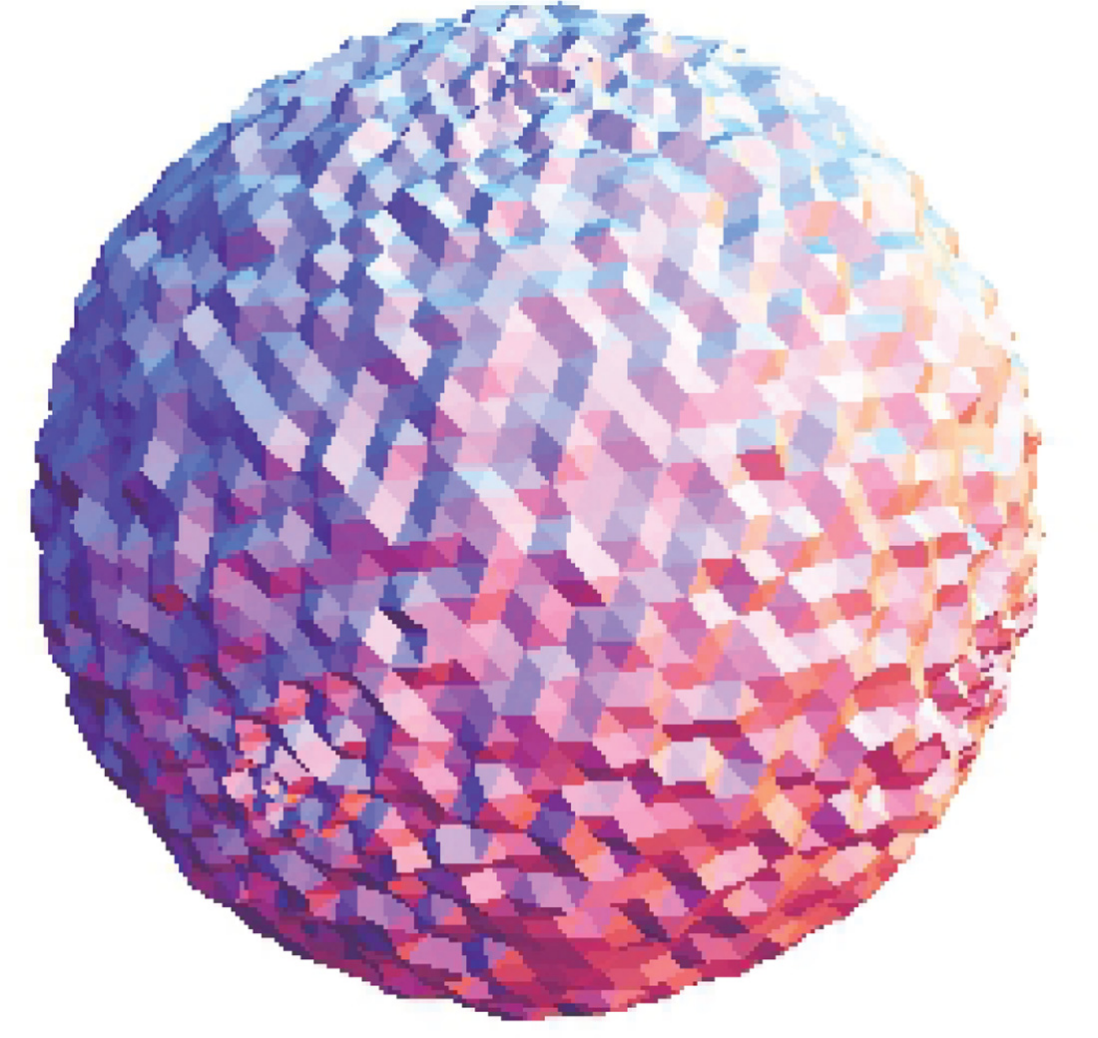
The sphere point set model with a noise level of 0.50.

The Stanford bunny and bimba point set model with a noise level of 0.25 is selected to test the proposed smoothing algorithm because they contain sharp features and are corrupted by a lower level of noise. Observations are also recorded for the higher noise level of 0.50. It is essential for the smoothing algorithm to preserve the sharp edges and retain the structure of the model when carrying out the denoising procedure. The sphere point set model with a noise level of 0.25 and 0.50 is also considered and tested because it contains no features. The proposed smoothing algorithm modifies the position of the point set to achieve smoothing without altering the existing point connectivity. The noise-free point set models are shown in Figs 1–3 whereas models with noise levels of 0.25 and 0.50 are shown in Figs 4–9. In this study, although the Stanford bunny point set model contains a total of 11146 data points; only 1858 data points are uniformly selected. The Bimba point set model, which contains a total of 74764 data points; only 12461 data points are uniformly selected. The sphere point set model, which contains a total of 4098 data points; only 683 data points are uniformly selected. In another words, for these three point set models, approximately 16.7% of the total points, are uniformly selected for both noise levels to carry out the denoising process by the proposed algorithm.

For noise levels of 0.25 and 0.50, the user-defined values for *B*, *k*_1_, and *k*_2_ are 50, 10, and 20, respectively. These user-defined values are the same for the three point set models. To reiterate, the optimum smoothing parameter is obtained from our method, as detailed in [17]. For Stanford bunny point set model, in Step 5, the obtained optimum smoothing parameter *ρ* for the 10- and 20-nearest neighborhoods is the same, 0.000003, for noise level 0.25, whereas for noise level 0.50, the obtained optimum smoothing parameter *ρ* for the 10- and 20-nearest neighbourhoods is 0.00005 and 0.00001, respectively. For bimba point set model, in Step 5, the obtained optimum smoothing parameter *ρ* for the 10- and 20-nearest neighborhoods is 0.0001 and 0.00007, respectively for noise level 0.25, whereas for noise level 0.50, the obtained optimum smoothing parameter *ρ* for the 10- and 20-nearest neighbourhoods is 0.0025 and 0.0007, respectively. For sphere point set model, in Step 5, the obtained optimum smoothing parameter *ρ* for the 10- and 20-nearest neighborhoods are the same, that is 0.03 for noise level 0.25, whereas for noise level 0.50, the obtained optimum smoothing parameter *ρ* for the 10- and 20-nearest neighbourhoods is 0.3 and 0.5, respectively.

**Fig 10:**
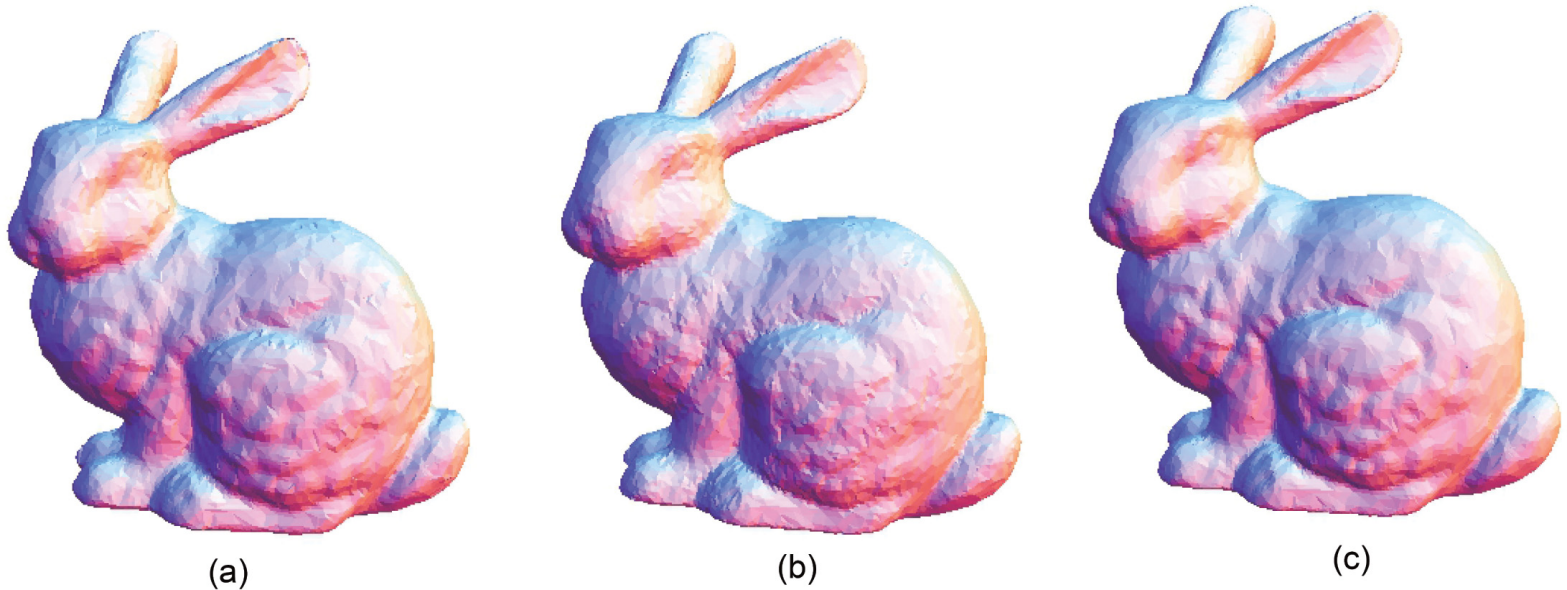
Comparison for the smoothing result from the Stanford bunny point set model with a noise level of 0.25. (a) Proposed smoothing algorithm. (b) HC Laplacian smoothing algorithm. (c) Algebraic point set surface smoothing algorithm.

**Fig 11:**
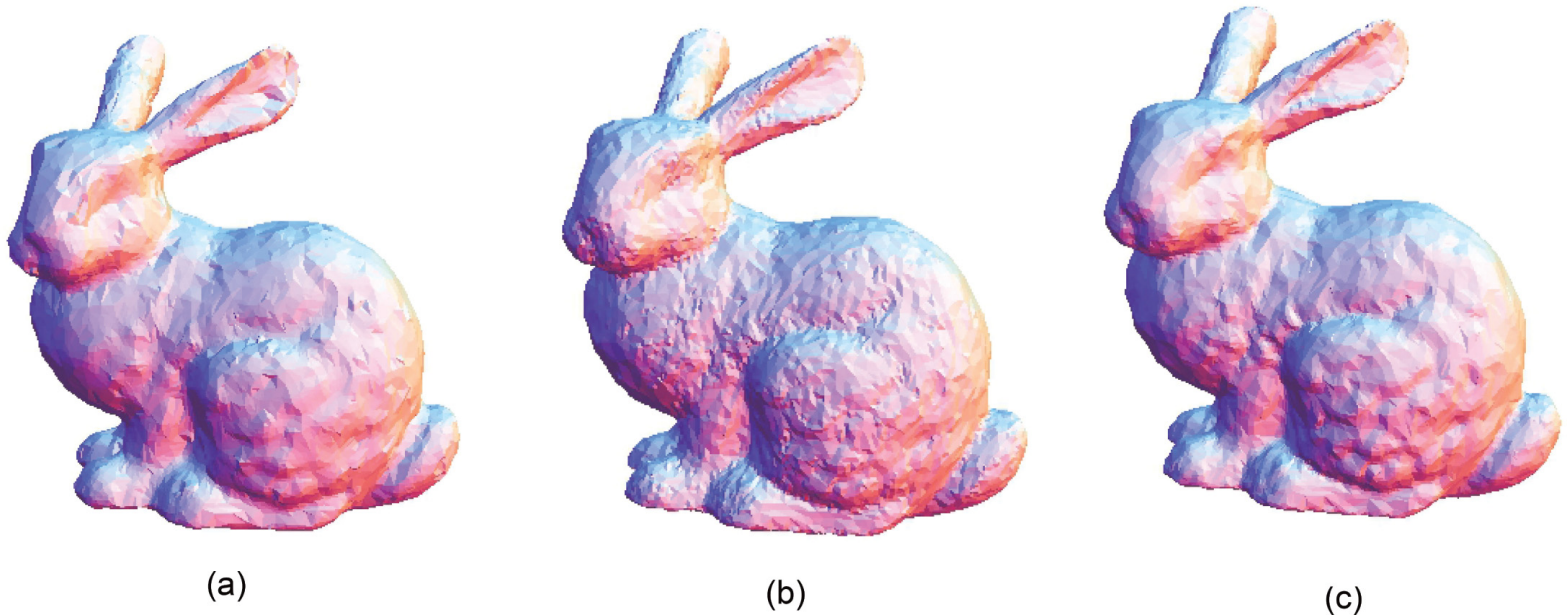
Comparison for the smoothing result from the Stanford bunny point set model with a noise level of 0.50. (a) Proposed smoothing algorithm. (b) HC Laplacian smoothing algorithm. (c) Algebraic point set surface smoothing algorithm.

**Fig 12:**
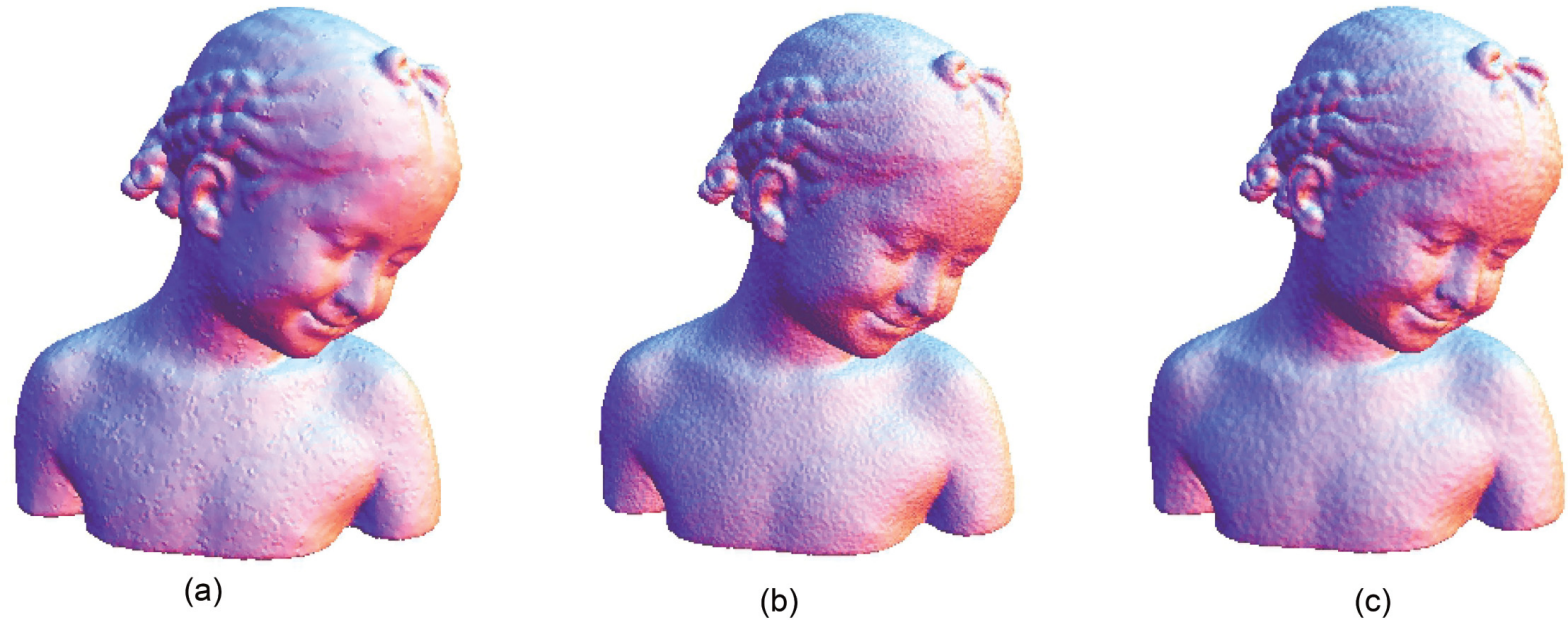
Comparison for the smoothing result from the bimba point set model with a noise level of 0.25. (a) Proposed smoothing algorithm. (b) HC Laplacian smoothing algorithm. (c) Algebraic point set surface smoothing algorithm.

**Fig 13:**
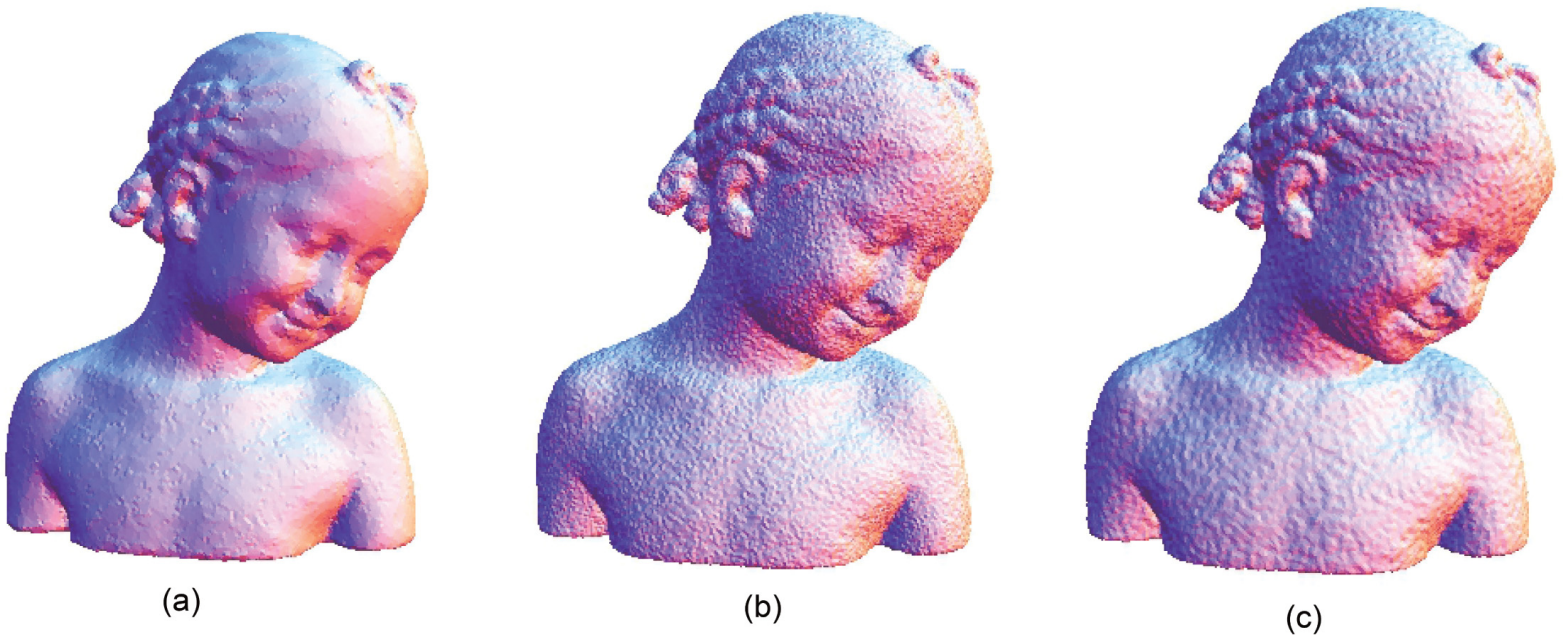
Comparison for the smoothing result from the bimba point set model with a noise level of 0.50. (a) Proposed smoothing algorithm. (b) HC Laplacian smoothing algorithm. (c) Algebraic point set surface smoothing algorithm.

**Fig 14:**
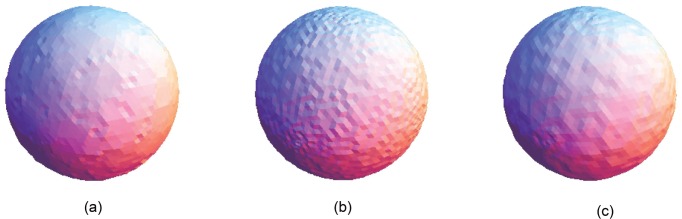
Comparison for the smoothing result from the sphere point set model with a noise level of 0.25. (a) Proposed smoothing algorithm. (b) HC Laplacian smoothing algorithm. (c) Algebraic point set surface smoothing algorithm.

**Fig 15:**
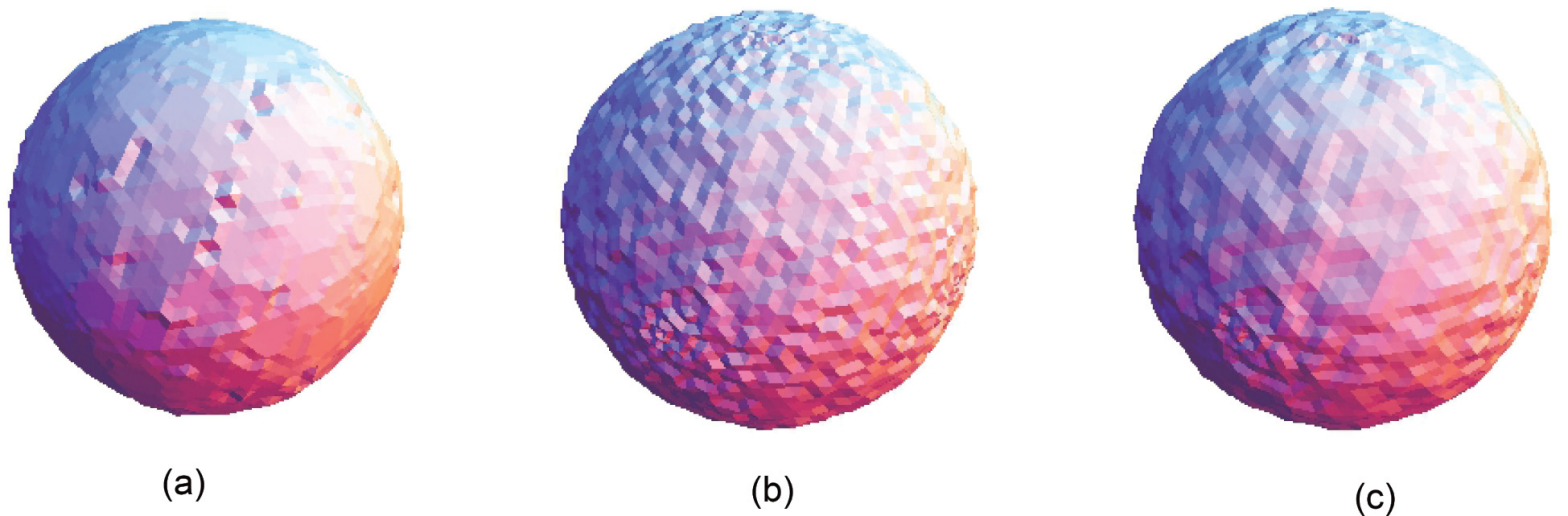
Comparison for the smoothing result from the sphere point set model with a noise level of 0.50. (a) Proposed smoothing algorithm. (b) HC Laplacian smoothing algorithm. (c) Algebraic point set surface smoothing algorithm.

The local *h* value acts as an indicator for the distribution of the data point in a neighbourhood, that is, the average nearest distance of points. This implies that if the local *h* value is higher than the global *h* value, the average nearest distance of points in that particular neighbourhood is further apart, by average comparison. We assume that the region has the possibility of being contaminated by the noise or contains a featured area. Otherwise, it can be considered a smooth region. Therefore, with this assumption, *k*_1_ is used when the local *h* is greater than the global *h*; otherwise, a higher value of *k*_2_ is used for the opposite condition. Smaller values of *k*_1_ and *k*_2_ are chosen because the proposed algorithm can achieve better denoising results while preserving features. Principal component analysis (PCA) is used to estimate the normal vector of a particular neighbourhood because it is required to carry out the surface patch fitting procedure before projecting the data points onto the thin-plate spline surface patch. The number of data points *S* that is uniformly selected is another issue that requires attention. When a greater number of data points *S* is selected, the sharp features will be smoothed out, whereas for a lower number of data points *S*, the noisy data will not be successfully denoised. The proposed smoothing algorithm produces the results shown in Figs 10a, 11a, 12a, 13a, 14a and 15a based on the optimum conditions described in this section.

The obtained result from the proposed algorithm is compared with that of the HC mesh smoothing algorithm, as shown in Figs 10b, 11b, 12b, 13b, 14b and 15b. The main difference between these algorithms is iteration. The proposed algorithm is a non-iterative procedure that is a one-step smoothing procedure, whereas the HC smoothing algorithm is an iterative procedure. The disadvantage of iteration in the HC smoothing algorithm is that features are slightly smoothed out, whereas the proposed smoothing algorithm preserves features. In comparison with the algebraic MLS, as shown in Figs 10c, 11c, 12c, 13c, 14c and 15c, those point set models with features are slightly better preserved compared to the proposed approach. However, the feature preservation by the proposed algorithm is comparable to that of algebraic MLS visually. The smoothing results for the noisy sphere point set models by using the proposed approach are also comparable to the algebraic MLS visually. In addition, there is a trade-off between performance and point set density in the algebraic MLS, but this is not an issue in the proposed algorithm because the data points are only partially selected for the smoothing procedure. Note that the default parameters of HC smoothing algorithm and algebraic MLS that are available in MeshLab are being used throughout the comparison.

## Conclusion

In this paper, a point set smoothing algorithm, based on a bootstrap thin-plate spline surface, has been presented. It is a simple algorithm that incorporates a statistical method into smoothing the noisy point set model and preserves the features of the model. In the proposed algorithm, only the uniformly selected data points are smoothed instead of all points in the point set. It is hoped that the proposed smoothing algorithm can contribute to the growing body of literature on the point set smoothing method.

## Supporting Information

S1 Dataset Noise-free Stanford Bunny. Data is used in preparation of Fig 1.
(OFF)

S2 Dataset Stanford Bunny with a Noise Level of 0.25. Data is used in preparation of Fig 4.
(OFF)

S3 Dataset Stanford Bunny with a Noise Level of 0.50. Data is used in preparation of Fig 5.
(OFF)

S4 Dataset Noise-free Bimba. Data is used in preparation of Fig 2.
(OFF)

S5 Dataset Bimba with a Noise Level of 0.25. Data is used in preparation of Fig 6.
(OFF)

S6 Dataset Bimba with a Noise Level of 0.50. Data is used in preparation of Fig 7.
(OFF)

S7 Dataset Noise-free Sphere. Data is used in preparation of Fig 3.
(OFF)

S8 Dataset Sphere with a Noise Level of 0.25. Data is used in preparation of Fig 8.
(OFF)

S9 Dataset Sphere with a Noise Level of 0.50. Data is used in preparation of Fig 9.
(OFF)
